# Risk Factors of Velamentous Cord Insertion in Singleton Pregnancies—A Systematic Review and Meta-Analysis

**DOI:** 10.3390/jcm13185551

**Published:** 2024-09-19

**Authors:** Antonios Siargkas, Ioannis Tsakiridis, Athanasios Gatsis, Catalina De Paco Matallana, Maria Mar Gil, Petya Chaveeva, Themistoklis Dagklis

**Affiliations:** 1 Third Department of Obstetrics and Gynecology, School of Medicine, Faculty of Health Sciences, Aristotle University of Thessaloniki, Agiou Dimitriou, 54124 Thessaloniki, Greece; iotsakir@gmail.com (I.T.); athanasiosgatsis@gmail.com (A.G.); themistoklisdagklis@gmail.com (T.D.); 2 Institute for Biomedical Research of Murcia, IMIB-Arrixaca, El Palmar, Faculty of Medicine, Universidad de Murcia, 30120 Murcia, Spain; katydepaco@gmail.com; 3 Maternal Fetal Medicine Unit, Department Obstetrics and Gynecology, Virgen de la Arrixaca, 30120 Murcia, Spain; 4 School of Medicine, Universidad Francisco de Vitoria, 28223 Madrid, Spain; mariadelmar.gil@ufv.es; 5 Department of Obstetrics and Gynecology, Hospital Universitario de Torrejón, 28850 Madrid, Spain; 6 Ultrasound and Fetal Medicine Unit, Obstetrics and Gynecology Department, Hospital Universitario La Paz, 28046 Madrid, Spain; 7 Department of Obstetrics and Gynecology, Faculty of Medicine, Medical University of Pleven, 5800 Pleven, Bulgaria; chaveevapetya@gmail.com; 8 Fetal Medicine Unit, Dr. Shterev Hospital, 1330 Sofia, Bulgaria

**Keywords:** assisted reproductive technology, nulliparity, placenta previa, smoking, chronic hypertension, abnormal cord insertion

## Abstract

**Objective:** This meta-analysis aims to quantitatively summarize current data on various potential risk factors of velamentous cord insertion (VCI). A better understanding of these risk factors could enhance prenatal identification both in settings with routine screening and in those where universal screening for cord insertion anomalies is not yet recommended. **Methods:** A systematic search was conducted in MEDLINE, Cochrane Library, and Scopus from their inception until 7 February 2024. Eligible studies included observational studies of singleton pregnancies with VCI, identified either prenatally or postnatally, compared with pregnancies with central or eccentric cord insertion. Analyses were performed using DerSimonian and Laird random-effects models, with outcomes reported as risk ratios (RR) or mean differences with 95% confidence intervals (CI). **Results:** In total, 14 cohort and 4 case-control studies were included, reporting on 952,163 singleton pregnancies. Based on the cohort studies, the overall prevalence of VCI among singleton pregnancies was calculated to be 1.54%. The risk of VCI was significantly higher among pregnancies conceived using assisted reproductive technology (RR, 2.32; 95% CI: 1.77–3.05), nulliparous women (RR, 1.21; 95% CI: 1.15–1.28), women who smoked (RR, 1.14; 95% CI: 1.08–1.19), and pregnancies diagnosed with placenta previa (RR, 3.60; 95% CI: 3.04–4.28). **Conclusions:** This meta-analysis identified assisted reproductive technology, nulliparity, smoking, and placenta previa as significant risk factors of VCI among singleton pregnancies. These findings could inform screening policies in settings where universal screening for cord insertion is not routinely performed, suggesting a targeted approach for women with these specific risk factors.

## 1. Introduction

**Figure 1:**
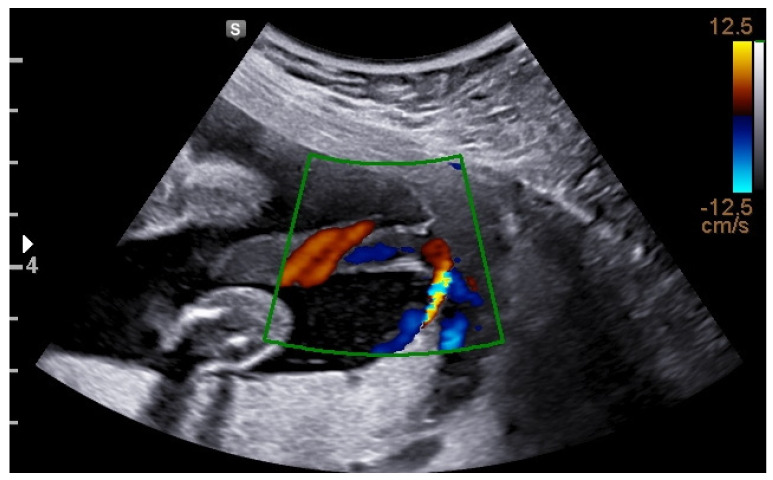
Ultrasound image depicting a velamentous cord insertion.

In a velamentous cord insertion (VCI), the umbilical cord inserts into the fetal membranes (between the amnion and the chorion) away from the placental margin, and the vessels traverse between these membranes before reaching the placenta, as depicted in Figure 1 [1]. In cases of VCI, there may be an absence of the protective effect of Wharton’s jelly, normally present around the vessels [2]. Placentas with non-central insertions may be less effective in supporting fetal growth despite their normal or even increased size. This decreased placental efficiency may be explained by a relative reduction in the chorionic vascular density of the placenta, as the cord is displaced from the center [3]. The reported occurrence of VCI among singleton pregnancies is 1.4%, and it was associated with several adverse perinatal outcomes including stillbirth, pre-eclampsia, placental abruption, small-for-gestational-age neonates, preterm delivery, emergency cesarean section (CS), reduced Apgar score and higher admission rate to the neonatal intensive care unit [4].

These findings, along with the feasibility of antenatal recognition of VCI, underscore the importance of a more systematic diagnostic approach; studies have shown that second-trimester sonographic identification of VCI is accurate, with an exceptionally high specificity of close to 100% but a lower sensitivity of about 70% [5]. Current recommendations vary regarding the need to identify a VCI antenatally. Thus, the American Institute of Ultrasound in Medicine recommends documenting abnormal cord insertions [6], whereas the International Society of Ultrasound in Obstetrics and Gynecology advises that an umbilical cord insertion assessment during mid-trimester scan is optional; however, an incidental finding of VCI should be documented [7]. 

A comprehensive understanding of risk factors for VCI may improve prenatal identification in settings with routine screening but also in settings where universal screening for cord insertion anomalies is not yet recommended and a targeted approach for women with specific risk factors should at least be considered. Published data have identified a variety of potential risk factors for VCI in singleton pregnancies, i.e., advanced maternal age, previous history of CS, Caucasian ethnicity, use of assisted reproductive technology (ART), smoking, placenta previa, nulliparity and chronic hypertension [8,9,10]. This meta-analysis aimed to conduct a rigorous evaluation and statistical analysis of the current evidence on possible risk factors for VCI. 

## 2. Methods

This meta-analysis adhered to the Preferred Reporting Items for Systematic reviews and Meta-Analyses (PRISMA) [11] and Meta-analysis Of Observational Studies in Epidemiology (MOOSE) guidelines [12] and was registered with the International Prospective Register of Systematic Reviews (PROSPERO) with the protocol number: CRD42024512296. Given its nature of synthesizing data from previously published literature, the study was exempt from the need for ethical approval and patient consent.

### 2.1. Search Strategy

The research question guiding this systematic search was, “Which population characteristics could serve as indicators of increased risk for VCI in singleton pregnancies”. We crafted a search strategy employing keywords such as “umbilical cord insertion”, “cord insertion”, “insertion of the cord”, “placental cord insertion”, “velamentous”, “abnormal” and “aberrant”. The details of the search strategy can be found in the Supplementary Materials. We searched MEDLINE, Scopus and Cochrane databases from their inception until 7 February 2024. The identified records were managed using Rayyan (Rayyan Systems Inc., Cambridge, MA, USA), a web-based reference management tool. Further, we examined references from relevant articles and conducted manual searches online to identify additional studies. After removing duplicates, we screened titles and abstracts to exclude studies not pertinent to our question and then thoroughly reviewed the full texts of the remaining articles to determine their inclusion. This process was independently carried out by two reviewers (A.S. and A.G, both doctors) blind to each other’s selections, with disagreements resolved through discussion or, if necessary, by a third reviewer (I.T., biostatistician).

### 2.2. Selection Criteria

Observational studies written in English, examining possible risk factors and population characteristics in singleton pregnancies identified with VCI either prenatally or following delivery, were considered eligible. The comparison group included pregnancies with central/eccentric cord insertion (CCI). In studies where the control groups included pregnancies with all types of non-velamentous umbilical cord insertions, adjustments were made, when possible, to ensure comparisons were exclusively with pregnancies having CCI. If such adjustments were not feasible, the studies were excluded. When the same database was utilized across two or more studies covering overlapping periods, we exclusively utilized data from the study encompassing the largest population. Raw data on perinatal outcomes were required. Abstracts and unpublished studies were not included.

### 2.3. Data Extraction

A standardized data collection template was prepared ahead of the study selection. This template captured study characteristics such as author, publication year, journal, study location, methodology, criteria for inclusion and exclusion, timing and definition of VCI diagnosis, study demographics and investigated risk factors. A second part of the template was dedicated to collecting data on predetermined outcomes, including raw data and, where available, adjusted odds ratios or adjusted risk ratios. Our protocol specified that outcomes reported in at least three studies would be considered for analysis, even if not initially outlined. We reached out to authors for missing data or clarifications and selected the most comprehensive report for studies with multiple publications on the same cohort.

### 2.4. Outcomes of Interest

Outcomes of interest included every risk factor reported by three or more studies, including ART, maternal age, prior CS, smoking, placenta previa, nulliparity, chronic hypertension and any other possible risk factor with adequate data as stipulated by our protocol.

### 2.5. Quality and Bias Assessment

The quality of the included studies was independently assessed by two researchers (A.S. and A.G) using the Newcastle−Ottawa scale [13], which evaluates the selection of the study groups, comparability of the groups, and ascertainment of the outcome/risk factor, employing a star system that assigns up to 9 points for high quality. Additionally, the Quality In Prognosis Studies (QUIPS) tool [14] was used to evaluate the risk of bias across six domains: study participation, attrition, prognostic factor measurement, outcome measurement, study confounding and statistical analysis/reporting, with studies rated on a three-point scale (low, moderate, high). Discrepancies in the assessment of quality or bias were reviewed and resolved by a third reviewer (I.T.).

### 2.6. Statistical Analysis

In our primary analysis, the cumulative raw data were analyzed to calculate the various risk factors’ effect on the VCI prevalence. This data synthesis involved computing effect sizes and their 95% confidence intervals (CI) via Review Manager software, version 5.4.1. We determined risk ratios (RR) for binary outcomes using the Mantel–Haenszel technique and mean differences (MD) for continuous variables through the inverse variance method. Due to the significant variability in observational studies, we followed the Cochrane Handbook’s recommendation to employ the DerSimonian and Laird random-effects model as the default analytical method. To evaluate the heterogeneity of the included studies, we utilized two approaches. The I^2^ statistic was employed to quantify the proportion of the total variance in the observed effect sizes that was due to differences between studies rather than chance. I^2^ values of up to 40% might be unimportant, 30–60% moderate, 50–90% substantial and 75–100% considerable [15]. Additionally, we applied the Cochran Q test to examine the homogeneity of the effect sizes across studies, with a *p*-value threshold of 0.10 for statistical significance. In R version 2.15.1 (R Foundation for Statistical Computing, Vienna, Austria) [16], the package meta [17] was employed to generate the funnel plot and the package dmetar [18] to perform the Egger’s test. These methods were used to assess publication bias only for the outcome with the higher number of published studies.

### 2.7. Sensitivity Analysis

As per our established methodology, we carried out a sensitivity analysis that only included cases identified prenatally. This was based upon the premise that our end goal is a better and more focused prenatal diagnosis of VCI. Furthermore, we conducted another sensitivity review that considered only those studies deemed as having a low or moderate risk of bias, as per the QUIPS tool criteria. The objective here was to filter the data less likely to be affected by bias and verify if these refined results would align with our initial findings. Notably, a sensitivity analysis was only deemed feasible if data from three or more studies were available for evaluation.

## 3. Results

**Figure 2:**
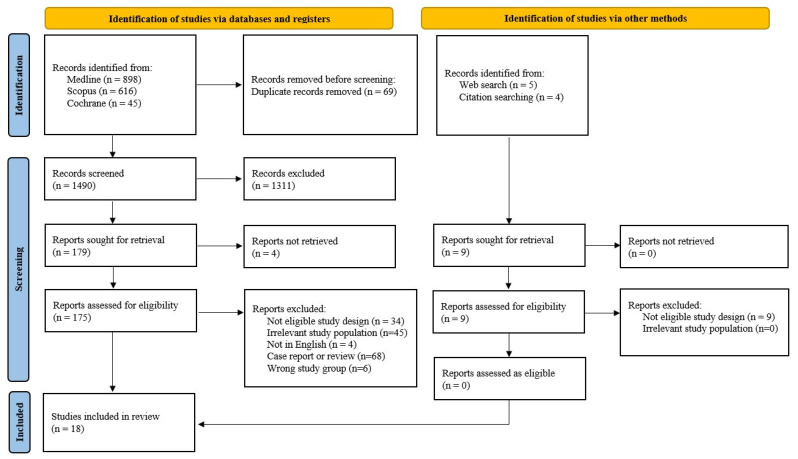
Study selection flow diagram.

Initially, 1559 records were identified from the Medline, Scopus, and Cochrane databases, while other methods like Web search and citation searching contributed nine additional records. After removing 69 duplicates, we screened 1490 records and excluded 1311 for various reasons. Upon assessing for eligibility, we evaluated 175 full texts, and 18 studies met the eligibility criteria for the review. The other search methods resulted in nine relevant reports, none of which was eligible (Figure 2). The two reviewers achieved an excellent coefficient of agreement on article selection (Cohen’s kappa, 0.932), resulting in the retention of 14 cohort [8,9,10,19,20,21,22,23,24,25,26,27,28,29] and 4 case-control studies [30,31,32,33] for the final analysis. The characteristics of the included studies are presented in Table 1.

### 3.1. Quality and Risk of Bias Assessment of the Studies

The quality of the studies assessed using the Newcastle–Ottawa Scale varied, with six achieving the top score of nine stars [8,9,19,20,24,26], indicating excellent methodology. Five studies earned eight stars [23,25,27,29,32] and five received seven stars [10,21,22,28,33], reflecting solid research approaches, while two studies scored six stars [30,31], suggesting some methodological concerns. The commonest weakness of the studies was the comparability category. No other significant deficits were noted (Table 2). A risk of bias visualization according to QUIPS was constructed for each study next to every forest plot. The QUIPS tool’s domains were each assigned a corresponding letter, ranging from A for study participation, B for study attrition, C for the measurement of prognostic factors, D for outcome measurement, E for study confound, and F for statistical analysis and reporting. This systematic approach facilitated a comprehensive evaluation of each study’s methodological quality and potential biases.

### 3.2. Raw Data Analysis

We included 14 relevant cohort studies and, based on their data (951,343 singleton pregnancies), the prevalence of VCI was calculated to be 1.54% (95% CI 1.52% to 1.57%).

### 3.3. Risk Factors’ Analyses

**Figure 3:**
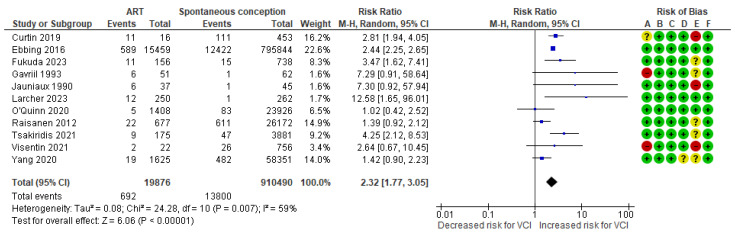
Forest plot demonstrating the risk for VCI in singleton pregnancies relative to the use of ART. Abbreviations: ART, assisted reproductive technology; CI, confidence interval; M−H, Mantel−Haenszel method; VCI, velamentous cord insertion.

In a composite analysis of eight cohort [9,10,20,24,25,26,27,29] and three case-control [30,32,33] studies, 692 cases of VCI were reported among the ART group, accounting for approximately 3.48% of the pregnancies, while the control group had 13,800 VCI cases, representing approximately 1.52% of the pregnancies. The occurrence of VCI in pregnancies with ART was significantly higher than those in the control group, with an RR of 2.32 (95% CI 1.77 to 3.05). Substantial heterogeneity was observed across the studies (*p* = 0.007; I^2^ = 59%) (Figure 3).

**Figure 4:**
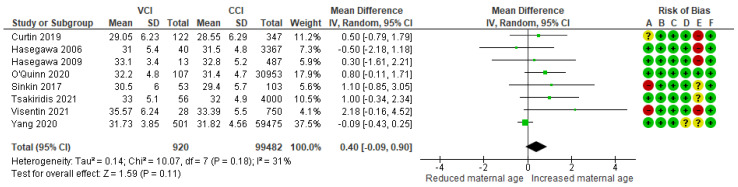
Forest plot demonstrating the risk for VCI in singleton pregnancies relative to mean maternal age. Abbreviations: CCI, central/eccentric cord insertion; CI, confidence interval; IV, weighted mean difference; SD, standard deviation; VCI, velamentous cord insertion.

In a composite analysis of six cohort [9,10,21,22,25,26] and two case-control [30,31] studies, the mean maternal age was assessed. The mean difference in maternal age between the VCI and CCI groups was +0.40 (95% CI −0.09 to 0.90)—not significantly different. There was low-to-moderate heterogeneity observed across the studies (*p* = 0.18; I^2^ = 31%) (Figure 4).

**Figure 5:**
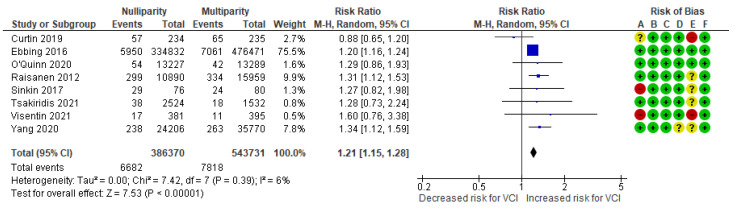
Forest plot demonstrating the risk for VCI in singleton pregnancies relative to parity. Abbreviations: CI, confidence interval; M−H, Mantel−Haenszel method; VCI, velamentous cord insertion.

In a composite analysis of six cohort [9,10,20,24,25,26] and two case-control [30,31] studies, 6682 cases of VCI were reported among the pregnancies of nulliparous women, accounting for approximately 1.73% of the pregnancies, while the control group had 7818 cases, representing approximately 1.44% of the pregnancies. The occurrence of VCI among nulliparous women was significantly higher compared to those in the multiparous group, with an RR of 1.21 (95% CI 1.15 to 1.28). There was low heterogeneity observed across the studies (*p* = 0.39; I^2^ = 6%) (Figure 5).

**Figure 6:**
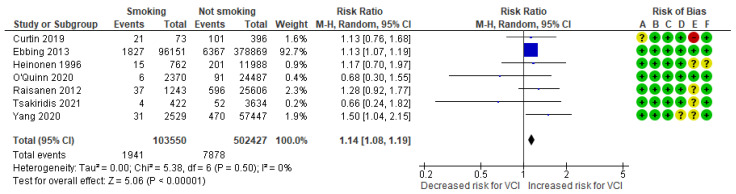
Forest plot demonstrating the risk for VCI in singleton pregnancies relative to smoking. Abbreviations: CI, confidence interval; M−H, Mantel−Haenszel method; VCI, velamentous cord insertion.

In a composite analysis of six cohort [8,9,23,24,25,26] and one case-control [30] study, 1941 cases of VCI were reported among the group of smokers, accounting for approximately 1.87% of the pregnancies, while the control group had 7878 cases, representing approximately 1.57% of the pregnancies. The occurrence of VCI among women who smoked was significantly different from those in the non-smoking group, with an RR of 1.14 (95% CI 1.08 to 1.19). No heterogeneity was observed across the studies (*p* = 0.50; I^2^ = 0%) (Figure 6).

**Figure 7:**
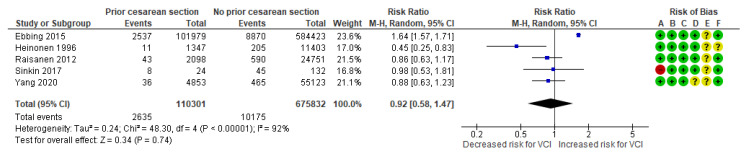
Forest plot demonstrating the risk for VCI in singleton pregnancies relative to having a prior cesarean section. Abbreviations: CI, confidence interval; M−H, Mantel−Haenszel method; VCI, velamentous cord insertion.

In a composite analysis of four cohort [19,23,24,26] and one case-control study [31], 2635 cases with VCI were reported among the group with prior CS, accounting for approximately 2.39% of the pregnancies, while the control group had 10,175 cases, representing approximately 1.51% of the pregnancies. The occurrence of VCI in pregnancies with prior CS was not significantly different from those in the control group, with an RR of 0.92 (95% CI 0.58 to 1.47). There was considerable heterogeneity observed across the studies (*p* < 0.001; I^2^ = 92%) (Figure 7).

**Figure 8:**
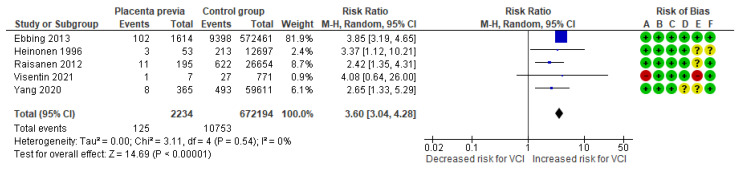
Forest plot of risk for VCI in singleton pregnancies relative to placenta previa. Abbreviations: CI, confidence interval; M−H, Mantel−Haenszel method; VCI, velamentous cord insertion.

In a composite analysis of five cohort studies [8,10,23,24,26], 125 cases of VCI were reported among the pregnancies complicated by placenta previa, accounting for approximately 5.59% of the pregnancies, while the control group had 10,753 cases of VCI, representing approximately 1.60% of the pregnancies. The occurrence of VCI in pregnancies with placenta previa was significantly different from those in the control group, with an RR of 3.60 (95% CI 3.04 to 4.28). No heterogeneity was observed across the studies (*p* = 0.54; I^2^ = 0%) (Figure 8).

**Figure 9:**
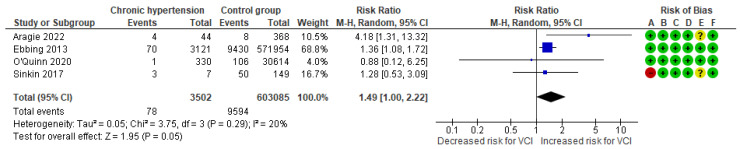
Forest plot demonstrating the risk for VCI in singleton pregnancies relative to chronic hypertension. Abbreviations: CI, confidence interval; M−H, Mante−Haenszel method; VCI, velamentous cord insertion.

In a composite analysis of three cohort [8,25,28] and one case-control study [31], 78 cases of VCI were reported among the women diagnosed with chronic hypertension, accounting for approximately 2.23% of the pregnancies, while the control group had 9594 cases, representing approximately 1.59% of the pregnancies. The occurrence of VCI in pregnancies with chronic hypertension was not significantly different from those in the control group, with an RR of 1.488 (95% CI 0.998 to 2.219). There was low heterogeneity observed across the studies (*p* = 0.29; I^2^ = 20%) (Figure 9).

**Figure 10:**
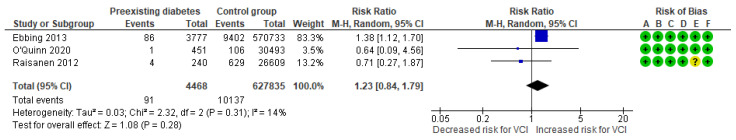
Forest plot demonstrating the risk for VCI in singleton pregnancies relative to pre-existing diabetes. Abbreviations: CI, confidence interval; M−H, Mante−Haenszel method; VCI, velamentous cord insertion.

In a composite analysis of three cohort studies [8,24,25], 91 cases of VCI were reported among pregnancies with pre-existing diabetes mellitus, accounting for approximately 2.03% of the pregnancies, while the control group had 10,137 cases, representing approximately 1.61% of the pregnancies. The occurrence of VCI in pregnancies of women with a diagnosis of diabetes was not significantly different from those in the control group, with an RR of 1.23 (95% CI 0.84 to 1.79). There was low heterogeneity observed across the studies (*p* = 0.31; I^2^ = 14%) (Figure 10).

The cumulative results of our primary analysis are compiled and displayed in Table 3.

### 3.4. Sensitivity Analyses Regarding Prenatal Diagnosis and Risk of Bias

Limiting our analysis to studies that provided data on prenatal diagnosis of umbilical cord insertion, we examined 18 studies, with 4 involving prenatal identification of VCI, and we could sufficiently assess three risk factors: ART, mean maternal age and nulliparity. The updated findings were consistent with the main results (Table 4 and Supplementary Figures S1–S3).

Subsequently, the studies identified as a high risk of bias were removed. Within the scope of ten analyses, seven were affected by the presence of high-risk bias studies. Following their exclusion, six out of seven analyses maintained comparable results to the initial findings. The updated findings were consistent with the main results (Table 4 and Supplementary Figures S4–S10).

### 3.5. Publication Bias

**Figure 11:**
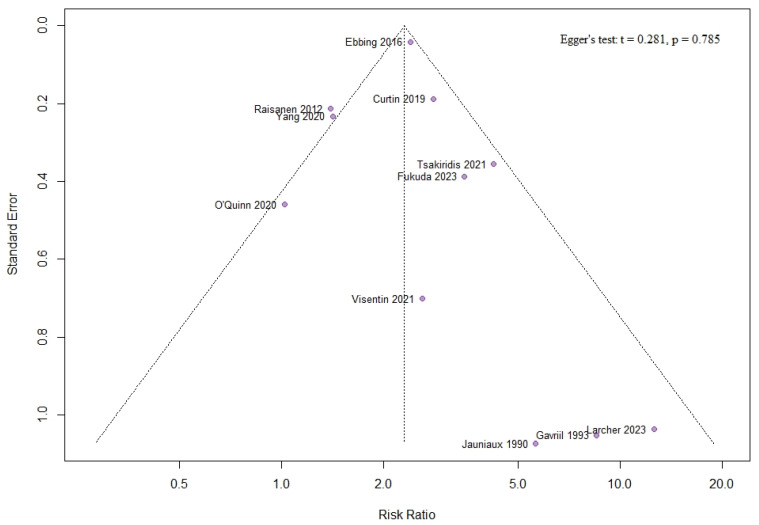
Funnel plot and Egger’s test regarding our most investigated outcome—assisted reproductive technology.

The risk factor with the most included studies was ART, which was tested for publication bias. Neither the funnel plot nor the Egger’s test demonstrated any indication of publication bias (Figure 11).

## 4. Discussion

### 4.1. Principal Findings

Our analysis found that first, the reported prevalence of VCI in singleton pregnancies is 1.54%, and second, the factors associated with an increased risk of VCI include ART, nulliparity, smoking and placenta previa.

### 4.2. Interpretation of the Findings

The largest investigated population was in the ART-VCI analysis, which incorporated 11 studies. The analysis demonstrated that ART is associated with a two-fold higher risk for VCI among singleton pregnancies and this association persisted in cases prenatally diagnosed with VCI and after excluding studies at high risk of bias. Our findings are consistent with those of a recent meta-analysis, which reported an OR of 2.14; however, this study included twin gestations in the analysis and the control group was broadly defined [34]. Our results support earlier epidemiological findings that pregnancies conceived via ART are associated with a higher incidence of umbilico-placental abnormalities [35,36,37]. The mechanism that ART could disrupt placentation has not been established; nevertheless, interventions such as controlled ovarian hyperstimulation, intrauterine insemination, gamete or embryo freezing, in-vitro fertilization, embryo culture, cell biopsy and blastocyst or embryo transfer may exercise oxidative, thermal, and mechanical stresses, and changes in DNA methylation that could alter the natural biological processes of reproduction [38,39]. Finally, related surgical procedures, such as septum excision, myomectomy, and other surgical treatments of uterine anomalies, may also contribute to the elevated risk of VCI, underscoring the need for further investigation in this area.

The analysis of maternal age in relation to the risk of VCI encompassed eight studies and revealed no significant increase in risk; only one study that categorized maternal age found that women aged over 35 years exhibited a higher risk of developing VCI (RR, 1.61) [23]. Moreover, two additional studies that were excluded from our meta-analysis due to the inclusion of cases with marginal cord insertion in the control group similarly identified maternal age above 35 years as a risk factor for VCI [40,41]. These findings suggest that the relationship between maternal age and VCI may be more nuanced, with significant risks manifesting particularly after the age of 35. This association could be partially attributed to the increased utilization of ART among this age group.

Nulliparity was identified as a significant risk factor for VCI, even when focusing on prenatally diagnosed pregnancies and studies with low risk of bias. While most small studies did not report a statistically significant association between nulliparity and VCI [9,10,25,30,31], the three larger studies [20,24,26], which made up over 90% of the pooled data, showed a strong association. No relevant studies explaining the pathophysiological cause were found, but we hypothesize that the lack of physiological adaptations that occur in the uterus and placenta during subsequent pregnancies may be a plausible explanation.

Regarding smoking, although most individual studies did not establish a significant relationship with VCI, the aggregated analysis indicated a 14% increased risk of VCI among smokers. Maternal smoking has been documented to adversely impact both the local immune response and microcirculation within decidual tissues [42]. It is conceivable that these alterations in the decidual environment during the implantation and the embryogenesis phase may play a role in the formation of VCI. A study of 83,708 women utilizing multiple regression models observed that exposure to fine particulate matter was positively associated with VCI and described two possible mechanisms: ischemia of the endometrium and intrauterine inflammation [43].

Prior CS was not associated with VCI in our meta-analysis. It seems that contrary to the low placental implantation, which is strongly associated with prior CS, abnormal cord insertion is not associated with them. However, placenta previa was identified as a significant risk factor for VCI, increasing its incidence fourfold [44].

Finally, no association was detected with chronic hypertension or pre-existing diabetes and VCI. However, it is noteworthy that chronic hypertension had a high RR of 1.49, indicating a potential 50% increase in the risk of VCI. The marginal lack of statistical significance is likely due to the sample size and the use of random effect models. Therefore, additional data are required to make a definitive conclusion about this matter.

### 4.3. Clinical and Research Implications

Currently, there is no consensus on the usefulness of universal screening for cord insertion anomalies. Therefore, our findings on the risk factors for VCI may inform the development of a more targeted screening approach for women exhibiting these risk factors. Given that isolated VCI is a primary risk factor for various adverse perinatal outcomes, including stillbirth [4], while also being the main risk factor for vasa previa [45]—a condition associated with significant perinatal mortality if prenatally undiagnosed [46] but preventable if diagnosed [47]—the importance of identifying VCI cannot be overstated [1,48]. Furthermore, recent studies have linked VCI with a twofold increased risk of cerebral palsy, suggesting that early detection of VCI could be crucial in identifying fetuses at a higher risk for this condition [49].

### 4.4. Strengths and Limitations

Our study’s main strength lies in its comprehensive design, which allowed us to include numerous studies and explore a wide array of possible risk factors. We maintained strict selection criteria, which led to more reliable estimates of effects and potentially reduced variability among the included studies. Our focus was to provide additional information on the prenatal diagnosis of VCI. To this end, our sensitivity analysis specifically targeting pregnancies with prenatal diagnosis of VCI may enhance the broader applicability of our findings.

The primary limitation of our meta-analysis stems from the nature of the included studies, all of which were observational, including case-control designs. Additionally, most of these studies focused primarily on the perinatal outcomes of VCI rather than investigating risk factors, making them susceptible to selection and recall biases. Furthermore, we were unable to examine the association between prenatally diagnosed VCI and all its risk factors, as not every study provided the necessary data. A further limitation is that in the studies with prenatally diagnosed cases, there was no systematic postnatal confirmation. Finally, none of the studies reported adjusted effect measures, preventing us from accounting for significant confounding variables. 

## 5. Conclusions

This meta-analysis identified ART, nulliparity, smoking and placenta previa as significant risk factors for VCI. These findings may assist the screening policy in settings where cord insertion is not universally offered. Additionally, this may enhance the antenatal detection of vasa previa, a condition that poses significant risks to pregnancies, as VCI is the primary risk factor for its development. Furthermore, the findings may also induce further high-quality research that addresses potential confounding variables to substantiate these associations. The exploration into the pathophysiological mechanisms underlying these relationships is imperative to enhance our understanding and further guide obstetric policies.

## Figures and Tables

**Table 1:** Characteristics of the included studies.

First Author, Year	Country	Study Period	Study Type	Inclusion Criteria	Exclusion Criteria	Cord Insertion Site Diagnosis	Risk Factors
Aragie et al., 2022 [28]	Ethiopia	2021	retrospective cohort	singleton pregnancies	marginal cord insertion, placenta specimens without intact umbilical cord, placenta with externally identifiable pathology, and bifurcated umbilical cord before its insertion	postnatally (inspection of cord insertion during the delivery)	chronic hypertension
Curtin et al., 2019 [30]	USA	2002–2015	retrospective case-control	singleton pregnancies	multiple pregnancies	postnatally (identification of cord insertion through pathology reports)	ART, maternal age, nulliparity, smoking, hypertension
Ebbing et al., 2013 [8]	Norway	1999–2009	retrospective cohort	singleton pregnancies delivered at 16–45 wk	multiple pregnancies	postnatally (form completed by the attending midwife or physician shortly after delivery)	smoking, pre-existing diabetes, placenta previa, chronic hypertension
Ebbing et al., 2015 [19]	Norway	1999–2011	retrospective cohort	singleton pregnancies delivered at 16–45 wk	multiple pregnancies	postnatally (inspection of cord insertion during the delivery)	prior CS
Ebbing et al., 2016 [20]	Norway	1999–2013	retrospective cohort	singleton pregnancies delivered at 16–45 wk	multiple pregnancies	postnatally (inspection of cord insertion during the delivery)	ART, nulliparity
Fukuda et al., 2023 [29]	Japan	2020–2022	retrospective cohort	singleton pregnancies	Intrauterine fetal death, multiple pregnancies, unknown method of conception, multiple-lobed and/or accessory placentas, undelivered placentas due to adhesion, and incomplete entry	postnatally (inspection of cord insertion during the delivery)	ART
Gavriil et al., 1993 [33]	Belgium		retrospective case-control	ART and intrauterine embryo transfer pregnancies	includes twin pregnancies but reports separate raw data for singleton pregnancies	postnatally through pathologic examination	ART
Hasegawa et al., 2006 [22]	Japan	2002–2004	retrospective cohort	singleton pregnancies	women who first visited the hospital after 20 weeks of gestation	postnatally (inspection of cord insertion during the delivery)	maternal age
Hasegawa et al., 2009 [21]	Japan	2005–2006	retrospective cohort	singleton pregnancies delivered at 22–41 wk	multiple pregnancies	postnatally (inspection of cord insertion during the delivery)	maternal age
Heinonen et al., 1996 [23]	Finland	1989–1993	retrospective cohort	singletons pregnancies delivered after 24 wk	multiple pregnancies and pregnancies with fetal chromosomal abnormalities or structural malformations	postnatally (inspection of cord insertion during the delivery)	prior CS, smoking, unmarried, placenta previa
Jauniaux et al., 1990 [32]	Belgium	1985–1988	retrospective case-control	singleton pregnancies conceived and delivered at term in ART clinic	multiple pregnancies and pregnancies with early vanishing twin phenomenon	postnatal macroscopic examination	ART
Larcher et al., 2023 [27]	Italy	2019–2021	prospective cohort	singleton pregnancies	prior CS-unmatched pregnancies and delivery at gestation age <32 weeks	prenatally (transvaginal/transabdominal) US examination at 11–14, 19–22 and 32–35 weeks	ART
O’Quinn et al., 2020 [25]	Canada	2012–2015	retrospective cohort	singleton pregnancies with completed anatomic survey, delivered >24^+6^ wk,	multiple pregnancies, placenta previa, vasa previa, no documented cord insertion type, or fetal anomalies	prenatally (at the 18–21-week anatomy ultrasound scan)	ART, maternal age, nulliparity, smoking, pre-existing diabetes, chronic hypertension
Raisanen et al., 2012 [24]	Finland	2000–2011	retrospective population-based registerstudy	singleton pregnancies, live-born and stillborn infants delivered afterthe 22 wk or weighing 500 g or more	multiple pregnancies	postnatally (inspection of cord insertion during the delivery)	ART, prior CS, pre-existing diabetes, smoking, placenta previa, nulliparity
Sinkin et al., 2017 [31]	USA	2005–2015	retrospective case-control	singleton pregnancies and at least one ultrasound examination by the maternal-fetal medicine service	fetal anomalies, gestational age at delivery <23 weeks, and vasa previa.	postnatally (according to placenta pathology records)	maternal age, maternal BMI, prior CS, nulliparity, chronic hypertension
Tsakiridis et al., 2021 [9]	Greece	2016–2020	retrospective cohort	singleton pregnancies with a second trimester anomaly scan at 20^+0^–23^+6^ wk	fetal abnormalities, vasa previa, and single umbilical artery	prenatally (anomaly scan at 20^+0^–23^+6^ wk)	ART, maternal age, nulliparity, smoking
Visentin et al., 2021 [10]	Italy	2016–2017	retrospective cohort	singleton pregnancies delivered >24 wk	multiple pregnancies, miscarriages and voluntary abortions.	postnatally (according to placenta pathology records)	ART, maternal age, nulliparity, placenta previa
Yang et al., 2020 [26]	China	2004–2014	retrospective cohort	singleton pregnancies	multiple pregnancies	prenatally (color Doppler ultrasonography)	ART, maternal age, nulliparity, smoking, prior CS, placenta previa

Abbreviations: ART, assisted reproductive technology; CS, cesarean section; wk, gestational week.

**Table 2:** Quality assessment of the included studies according to the Newcastle.

First Author, Year	Study Type	S1	S2	S3	S4	C	O1	O2	O3	Total
Aragie et al., 2022 [28]	retrospective cohort	b *	a *	b *	a *	-	b *	a *	a *	7
Curtin et al., 2019 [30]	retrospective case-control	b	a *	a *	a *	-	a *	a *	a *	6
Ebbing et al., 2013 [8]	retrospective cohort	a *	a *	a *	a *	a *, b *	b *	a *	a *	9
Ebbing et al., 2015 [19]	retrospective cohort	a *	a *	a *	a *	a *, b *	b *	a *	a *	9
Ebbing et al., 2016 [20]	retrospective cohort	a *	a *	a *	a *	a *, b *	b *	a *	a *	9
Fukuda et al., 2023 [29]	retrospective cohort	b *	a *	a *	a *	a *	b *	a *	a *	8
Gavriil et al., 1993 [33]	retrospective case control	a *	b	a *	a *	a *	b *	a *	a *	7
Hasegawa et al., 2006 [22]	retrospective cohort	b *	a *	a *	a *	-	b *	a *	a *	7
Hasegawa et al., 2009 [21]	retrospective cohort	b *	a *	a *	a *	-	b *	a *	a *	7
Heinonen et al., 1996 [23]	retrospective cohort	b *	a *	a *	a *	a *	b *	a *	a *	8
Jauniaux et al., 1990 [32]	retrospective case control	b *	a *	a *	a *	a *	a *	a *	a *	8
Larcher et al., 2023 [27]	prospective cohort	b *	a *	a *	a *	a *	b *	a *	a *	8
O’Quinn et al., 2020 [25]	retrospective cohort	a *	a *	a *	a *	b *	b *	a *	a *	8
Raisanen et al., 2012 [24]	retrospective cohort	b *	a *	a *	a *	a *, b *	b *	a *	a *	9
Sinkin et al., 2017 [31]	retrospective case-control	b	a *	a *	a *	-	a *	a *	a *	6
Tsakiridis et al., 2021 [9]	retrospective cohort	b *	a *	a *	a *	a *, b *	b *	a *	a *	9
Visentin et al., 2021 [10]	retrospective cohort	c	a *	a *	a *	a *	b *	a *	a *	7
Yang et al., 2020 [26]	retrospective cohort	a *	a *	a *	a *	a *, b *	b *	a *	a *	9

Abbreviations: a, first answer according to NOS; b, second answer according to NOS; c, third answer according to NOS; S, selection; C, comparability; O, outcome; *, attribution of a star according to NOS.

**Table 3:** Results of the meta-analysis regarding risk factors of velamentous cord insertion in singleton pregnancies.

Risk Factor	Number of Studies	VCI Cases/Total Cases (Exposed to Risk Factor)	VCI Cases/Total Cases (Not Exposed to Risk Factor)	RR	95% CI	I^2^; *p*-Value
Assisted reproductive technology	11	692/19,876 (3.48%)	13,800/910,490 (1.52%)	2.32	1.77–3.05	59%; 0.007
Maternal age	8	920 (VCI cases)	99,482 (CCI cases)	0.40 (MD)	−0.09 to 0.90	31%; 0.18
Nulliparity	8	6682/386,370 (1.73%)	7818/543,731 (1.44%)	1.21	1.15–1.28	6%; 0.39
Smoking	7	1941/103,550 (1.87%)	7878/502,427 (1.57%)	1.14	1.08–1.19	0%; 0.50
Prior cesarean section	5	2635/110,301 (2.39%)	10,175/675,832 (1.51%)	0.92	0.58–1.47	92%; <0.001
Placenta previa	5	125/2234 (5.59%)	10,753/672,194 (1.60%)	3.60	3.04–4.28	0%; 0.54
Chronic hypertension	4	78/3502 (2.23%)	9594/603,085 (1.59%)	1.488	0.998–2.219	20%; 0.29
Pre-existing diabetes	3	91/4468 (2.03%)	10,137/627,835 (1.61%)	1.23	0.84–1.79	14%; 0.31

Abbreviations: CI, confidence interval; I^2^ (Heterogeneity in meta-analysis); MD, mean difference; *p*-value, Cochran Q test’s *p*-value; RR, relative risk; VCI, velamentous cord insertion.

**Table 4:** Confidence intervals from all the analyses performed.

Risk Factor	Overall Analysis		Prenatally Diagnosed		RoB Sensitivity Analysis	
	RR	95% CI	RR	95% CI	RR	95% CI
Assisted reproductive technology	2.32	1.77–3.05	3.18	1.10–9.21	2.14	1.49–3.08
Maternal age	0.40	−0.09 to 0.90	0.40	−0.35 to 1.15	0.40	−0.35 to 1.15
Nulliparity	1.21	1.15–1.28	1.33	1.14–1.55	1.21	1.17–1.25
Smoking	1.14	1.08–1.19			1.15	1.05–1.26
Prior cesarean section	0.92	0.58–1.47			0.91	0.53–1.55
Placenta previa	3.60	3.04–4.28			3.56	2.94–4.30
Chronic hypertension	1.488	0.998–2.219			1.73	0.81–3.70

Abbreviations: CI, confidence interval; RR, relative risk; RoB, risk of bias.

## Data Availability

Not applicable.

## Supplementary Materials

The following supporting information can be downloaded at: https://www.mdpi.com/article/10.3390/jcm13185551/s1, Figure S1: Forest plot demonstrating the risk for prenatally diagnosed VCI in singleton pregnancies relative to the use of ART; Figure S2: Forest plot demonstrating the risk for prenatally diagnosed VCI in singleton pregnancies relative to mean maternal age; Figure S3: Forest plot demonstrating the risk for prenatally diagnosed VCI in singleton pregnancies relative to parity; Figure S4: Risk of bias sensitivity analysis regarding ART—VCI association in singleton pregnancies; Figure S5: Risk of bias sensitivity analysis regarding mean maternal age—VCI association in singleton pregnancies; Figure S6: Risk of bias sensitivity analysis regarding nulliparity—VCI association in singleton pregnancies; Figure S7: Risk of bias sensitivity analysis regarding smoking—VCI association in singleton pregnancies; Figure S8: Risk of bias sensitivity analysis regarding prior cesarean section—VCI association in singleton pregnancies; Figure S9: Risk of bias sensitivity analysis regarding placenta previa—VCI association in singleton pregnancies; Figure S10: Risk of bias sensitivity analysis regarding chronic hypertension—VCI association in singleton pregnancies.

## Abbreviations

ART	assisted reproductive technology
CCI	central/eccentric cord insertion
CI	confidence interval
CS	cesarean section
MD	mean difference
QUIPS	Quality In Prognosis Studies
RR	risk ratio
VCI	velamentous cord insertion

## References

[1] Jauniaux E., Ebbing C., Oyelese Y., Maymon R., Prefumo F., Bhide A. (2024). European association of perinatal medicine (EAPM) position statement: Screening, diagnosis and management of congenital anomalies of the umbilical cord. Eur. J. Obstet. Gynecol. Reprod. Biol..

[2] Sherer D.M., Al-Haddad S., Cheng R., Dalloul M. (2021). Current Perspectives of Prenatal Sonography of Umbilical Cord Morphology. Int. J. Women’s Health.

[3] Yampolsky M., Salafia C.M., Shlakhter O., Haas D., Eucker B., Thorp J. (2009). Centrality of the umbilical cord insertion in a human placenta influences the placental efficiency. Placenta.

[4] Siargkas A., Tsakiridis I., Pachi C., Mamopoulos A., Athanasiadis A., Dagklis T. (2023). Impact of velamentous cord insertion on perinatal outcomes: A systematic review and meta-analysis. Am. J. Obstet. Gynecol. MFM.

[5] Buchanan-Hughes A., Bobrowska A., Visintin C., Attilakos G., Marshall J. (2020). Velamentous cord insertion: Results from a rapid review of incidence, risk factors, adverse outcomes and screening. Syst. Rev..

[6] American Institute of Ultrasound in Medicine (2013). AIUM Practice Guideline for the Performance of Obstetric Ultrasound Examinations. J. Ultrasound Med..

[7] Coutinho C.M., Sotiriadis A., Odibo A., Khalil A., D’Antonio F., Feltovich H., Salomon L.J., Sheehan P., Napolitano R., Berghella V. (2022). ISUOG Practice Guidelines: Role of ultrasound in the prediction of spontaneous preterm birth. Ultrasound Obstet. Gynecol. Off. J. Int. Soc. Ultrasound Obstet. Gynecol..

[8] Ebbing C., Kiserud T., Johnsen S.L., Albrechtsen S., Rasmussen S. (2013). Prevalence, risk factors and outcomes of velamentous and marginal cord insertions: A population-based study of 634,741 pregnancies. PLoS ONE.

[9] Tsakiridis I., Dagklis T., Athanasiadis A., Dinas K., Sotiriadis A. (2022). Impact of Marginal and Velamentous Cord Insertion on Uterine Artery Doppler Indices, Fetal Growth, and Preeclampsia. J. Ultrasound Med. Off. J. Am. Inst. Ultrasound Med..

[10] Visentin S., Londero A.P., Santoro L., Pizzi S., Andolfatto M., Venturini M., Saraggi D., Coati I., Sacchi D., Rugge M. (2022). Abnormal umbilical cord insertions in singleton deliveries: Placental histology and neonatal outcomes. J. Clin. Pathol..

[11] Page M.J., Moher D., Bossuyt P.M., Boutron I., Hoffmann T.C., Mulrow C.D., Shamseer L., Tetzlaff J.M., Akl E.A., Brennan S.E. (2021). PRISMA 2020 explanation and elaboration: Updated guidance and exemplars for reporting systematic reviews. BMJ (Clin. Res. Ed.).

[12] Stroup D.F., Berlin J.A., Morton S.C., Olkin I., Williamson G.D., Rennie D., Moher D., Becker B.J., Sipe T.A., Thacker S.B. (2000). Meta-analysis of observational studies in epidemiology: A proposal for reporting. Meta-analysis Of Observational Studies in Epidemiology (MOOSE) group. JAMA.

[13] Wells G., Shea B., O’Connell D., Peterson J., Welch V., Losos M., Tugwell P. (2000). The Newcastle–Ottawa Scale (NOS) for Assessing the Quality of Non-Randomized Studies in Meta-Analysis.

[14] Hayden J.A., van der Windt D.A., Cartwright J.L., Côté P., Bombardier C. (2013). Assessing bias in studies of prognostic factors. Ann. Intern. Med..

[15] Higgins J., Thomas J., Chandler J., Cumpston M., Li T., Page M., Welch V. (2023). Cochrane Handbook for Systematic Reviews of Interventions Version 6.4.

[16] R Core Team (2013). R: A Language and Environment for Statistical Computing.

[17] Balduzzi S., Rücker G., Schwarzer G. (2019). How to perform a meta-analysis with R: A practical tutorial. Evid. -Based Ment. Health.

[18] Harrer M., Cuijpers P., Furukawa T., Ebert D.D. (2019). dmetar: Companion R Package for the Guide ‘Doing Meta-Analysis in R’. R Package Version 0.0.9000. http://dmetar.protectlab.org/.

[19] Ebbing C., Kiserud T., Johnsen S.L., Albrechtsen S., Rasmussen S. (2015). Third stage of labor risks in velamentous and marginal cord insertion: A population-based study. Acta Obstet. Et Gynecol. Scand..

[20] Ebbing C., Johnsen S.L., Albrechtsen S., Sunde I.D., Vekseth C., Rasmussen S. (2017). Velamentous or marginal cord insertion and the risk of spontaneous preterm birth, prelabor rupture of the membranes, and anomalous cord length, a population-based study. Acta Obstet. Et Gynecol. Scand..

[21] Hasegawa J., Matsuoka R., Ichizuka K., Kotani M., Nakamura M., Mikoshiba T., Sekizawa A., Okai T. (2009). Atypical variable deceleration in the first stage of labor is a characteristic fetal heart-rate pattern for velamentous cord insertion and hypercoiled cord. J. Obstet. Gynaecol. Res..

[22] Hasegawa J., Matsuoka R., Ichizuka K., Sekizawa A., Farina A., Okai T. (2006). Velamentous cord insertion into the lower third of the uterus is associated with intrapartum fetal heart rate abnormalities. Ultrasound Obstet. Gynecol. Off. J. Int. Soc. Ultrasound Obstet. Gynecol..

[23] Heinonen S., Ryynänen M., Kirkinen P., Saarikoski S. (1996). Perinatal diagnostic evaluation of velamentous umbilical cord insertion: Clinical, Doppler, and ultrasonic findings. Obstet. Gynecol..

[24] Räisänen S., Georgiadis L., Harju M., Keski-Nisula L., Heinonen S. (2012). Risk factors and adverse pregnancy outcomes among births affected by velamentous umbilical cord insertion: A retrospective population-based register study. Eur. J. Obstet. Gynecol. Reprod. Biol..

[25] O’Quinn C., Cooper S., Tang S., Wood S. (2020). Antenatal Diagnosis of Marginal and Velamentous Placental Cord Insertion and Pregnancy Outcomes. Obs. Gynecol.

[26] Yang M., Zheng Y., Li M., Li W., Li X., Zhang X., Wang R., Zhang J., Zhou F., Yang Q. (2020). Clinical features of velamentous umbilical cord insertion and vasa previa: A retrospective analysis based on 501 cases. Medicine.

[27] Larcher L., Jauniaux E., Lenzi J., Ragnedda R., Morano D., Valeriani M., Michelli G., Farina A., Contro E. (2023). Ultrasound diagnosis of placental and umbilical cord anomalies in singleton pregnancies resulting from in-vitro fertilization. Placenta.

[28] Aragie H., Kibret A.A., Teshager N.W., Adugna D.G. (2022). Velamentous cord insertion at the University of Gondar Comprehensive Specialized Hospital, Northwest Ethiopia. Clin. Epidemiol. Glob. Health.

[29] Fukuda E., Hamuro A., Kitada K., Kurihara Y., Tahara M., Misugi T., Nakano A., Tamaue M., Shinomiya S., Yoshida H. (2023). The Impact of Assisted Reproductive Technology on Umbilical Cord Insertion: Increased Risk of Velamentous Cord Insertion in Singleton Pregnancies Conceived through ICSI. Medicina.

[30] Curtin W.M., Hill J.M., Millington K.A., Hamidi O.P., Rasiah S.S., Ural S.H. (2019). Accuracy of fetal anatomy survey in the diagnosis of velamentous cord insertion: A case-control study. Int. J. Women’s Health.

[31] Sinkin J.A., Craig W.Y., Jones M., Pinette M.G., Wax J.R. (2018). Perinatal Outcomes Associated With Isolated Velamentous Cord Insertion in Singleton and Twin Pregnancies. J. Ultrasound Med..

[32] Jauniaux E., Englert Y., Vanesse M., Hiden M., Wilkin P. (1990). Pathologic features of placentas from singleton pregnancies obtained by in vitro fertilization and embryo transfer. Obstet. Gynecol..

[33] Gavriil P., Jauniaux E., Leroy F. (1993). Pathologic examination of placentas from singleton and twin pregnancies obtained after in vitro fertilization and embryo transfer. Pediatr. Pathol..

[34] Matsuzaki S., Ueda Y., Matsuzaki S., Nagase Y., Kakuda M., Lee M., Maeda M., Kurahashi H., Hayashida H., Hisa T. (2022). Assisted Reproductive Technique and Abnormal Cord Insertion: A Systematic Review and Meta-Analysis. Biomedicines.

[35] Yanaihara A., Hatakeyama S., Ohgi S., Motomura K., Taniguchi R., Hirano A., Takenaka S., Yanaihara T. (2018). Difference in the size of the placenta and umbilical cord between women with natural pregnancy and those with IVF pregnancy. J. Assist. Reprod. Genet..

[36] Ruiter L., Kok N., Limpens J., Derks J.B., de Graaf I.M., Mol B.W., Pajkrt E. (2015). Systematic review of accuracy of ultrasound in the diagnosis of vasa previa. Ultrasound Obstet. Gynecol. Off. J. Int. Soc. Ultrasound Obstet. Gynecol..

[37] Nagata C., Konishi K., Wada K., Tamura T., Goto Y., Koda S., Mizuta F., Iwasa S. (2019). Maternal Acrylamide Intake during Pregnancy and Sex Hormone Levels in Maternal and Umbilical Cord Blood and Birth Size of Offspring. Nutr. Cancer.

[38] Vrooman L.A., Xin F., Bartolomei M.S. (2016). Morphologic and molecular changes in the placenta: What we can learn from environmental exposures. Fertil. Steril..

[39] Furuya S., Kubonoya K., Yamaguchi T. (2021). Incidence and risk factors for velamentous umbilical cord insertion in singleton pregnancies after assisted reproductive technology. J. Obstet. Gynaecol. Res..

[40] Esakoff T.F., Cheng Y.W., Snowden J.M., Tran S.H., Shaffer B.L., Caughey A.B. (2015). Velamentous cord insertion: Is it associated with adverse perinatal outcomes?. J. Matern. Fetal Neonatal Med. Off. J. Eur. Assoc. Perinat. Med. Fed. Asia Ocean. Perinat. Soc. Int. Soc. Perinat. Obs..

[41] Eddleman K.A., Lockwood C.J., Berkowitz G.S., Lapinski R.H., Berkowitz R.L. (1992). Clinical significance and sonographic diagnosis of velamentous umbilical cord insertion. Am. J. Perinatol..

[42] Prins J.R., Hylkema M.N., Erwich J.J., Huitema S., Dekkema G.J., Dijkstra F.E., Faas M.M., Melgert B.N. (2012). Smoking during pregnancy influences the maternal immune response in mice and humans. Am. J. Obstet. Gynecol..

[43] Michikawa T., Morokuma S., Takeda Y., Yamazaki S., Nakahara K., Takami A., Yoshino A., Sugata S., Saito S., Hoshi J. (2022). Maternal exposure to fine particulate matter over the first trimester and umbilical cord insertion abnormalities. Int. J. Epidemiol..

[44] Santana E.F.M., Castello R.G., Rizzo G., Grisolia G., Araujo Júnior E., Werner H., Lituania M., Tonni G. (2022). Placental and Umbilical Cord Anomalies Diagnosed by Two- and Three-Dimensional Ultrasound. Diagnostics.

[45] Gross A., Markota Ajd B., Specht C., Scheier M. (2021). Systematic screening for vasa previa at the 20-week anomaly scan. Acta Obstet. Et Gynecol. Scand..

[46] Zhang W., Geris S., Al-Emara N., Ramadan G., Sotiriadis A., Akolekar R. (2021). Perinatal outcome of pregnancies with prenatal diagnosis of vasa previa: Systematic review and meta-analysis. Ultrasound Obstet. Gynecol. Off. J. Int. Soc. Ultrasound Obstet. Gynecol..

[47] Conyers S., Oyelese Y., Javinani A., Jamali M., Zargarzadeh N., Akolekar R., Hasegawa J., Melcer Y., Maymon R., Bronsteen R. (2024). Incidence and causes of perinatal death in prenatally diagnosed vasa previa: A systematic review and meta-analysis. Am. J. Obstet. Gynecol..

[48] Oyelese Y., Javinani A., Gudanowski B., Krispin E., Rebarber A., Akolekar R., Catanzarite V., D’Souza R., Bronsteen R., Odibo A. (2024). Vasa previa in singleton pregnancies: Diagnosis and clinical management based on an international expert consensus. Am. J. Obstet. Gynecol..

[49] Ebbing C., Rasmussen S., Kessler J., Moster D. (2023). Association of placental and umbilical cord characteristics with cerebral palsy: National cohort study. Ultrasound Obstet. Gynecol. Off. J. Int. Soc. Ultrasound Obstet. Gynecol..

