# Supplementary material for: Risk Factors of Velamentous Cord Insertion in Singleton Pregnancies—A Systematic Review and Meta-Analysis
Source: J Clin Med. 2024 Sep 19;13(18):5551. doi: 10.3390/jcm13185551 (PMC11432487; doi:10.3390/jcm13185551)
Supplement: Supplementary file 1 [file jcm-13-05551-s001.zip › jcm-3187466-supplementary.pdf]

**Figure S1.** Forest plot demonstrating the risk for prenatally diagnosed VCI in singleton pregnancies relative to the use of ART.

| | ART | | Spontaneous conception | | | Risk Ratio |
|---|---|---|---|---|---|---|
| Study or Subgroup | Events | Total | Events | Total | Weight | M-H, Random, 95% CI |
| Larcher 2023 | 12 | 250 | 1 | 262 | 17.3% | 12.58 [1.65, 96.01] |
| Tsakiridis 2021 | 9 | 175 | 47 | 3881 | 39.2% | 4.25 [2.12, 8.53] |
| Yang 2020 | 19 | 1625 | 482 | 58351 | 43.4% | 1.42 [0.90, 2.23] |
| **Total (95% CI)** | | **2050** | | **62494** | **100.0%** | **3.18 [1.10, 9.21]** |
| Total events | 40 | | 530 | | | |

Heterogeneity: Tau² = 0.62; Chi² = 9.79, df = 2 (P = 0.007); I² = 80%
Test for overall effect: Z = 2.13 (P = 0.03)

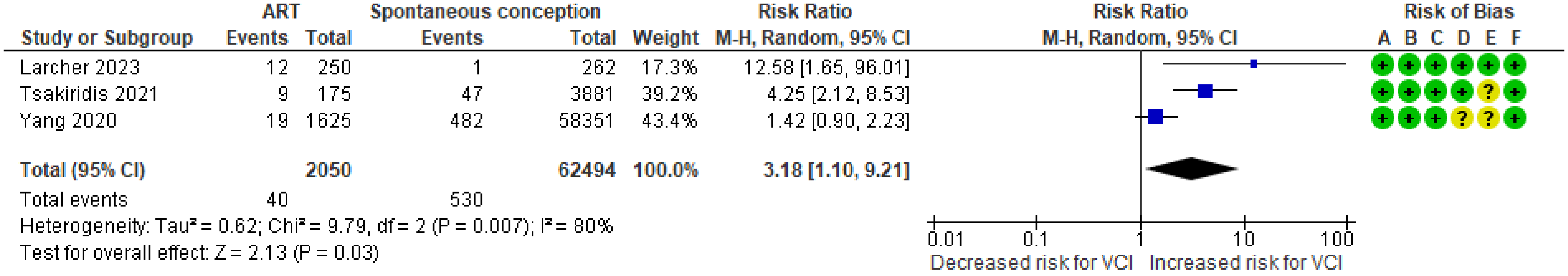


Abbreviations: ART, assisted reproductive technology; CI, confidence interval; M-H, Mantel–Haenszel method; VCI, velamentous cord insertion

**Figure S2.** Forest plot demonstrating the risk for prenatally diagnosed VCI in singleton pregnancies relative to mean maternal age.

| | VCI | | | CCI | | | | Mean Difference |
|---|---|---|---|---|---|---|---|---|
| Study or Subgroup | Mean | SD | Total | Mean | SD | Total | Weight | IV, Random, 95% CI |
| O'Quinn 2020 | 32.2 | 4.8 | 107 | 31.4 | 4.7 | 30953 | 30.5% | 0.80 [-0.11, 1.71] |
| Tsakiridis 2021 | 33 | 5.1 | 56 | 32 | 4.9 | 4000 | 20.0% | 1.00 [-0.34, 2.34] |
| Yang 2020 | 31.73 | 3.85 | 501 | 31.82 | 4.56 | 59475 | 49.5% | -0.09 [-0.43, 0.25] |
| **Total (95% CI)** | | | **664** | | | **94428** | **100.0%** | **0.40 [-0.35, 1.15]** |

Heterogeneity: Tau² = 0.27; Chi² = 5.16, df = 2 (P = 0.08); I² = 61%
Test for overall effect: Z = 1.04 (P = 0.30)

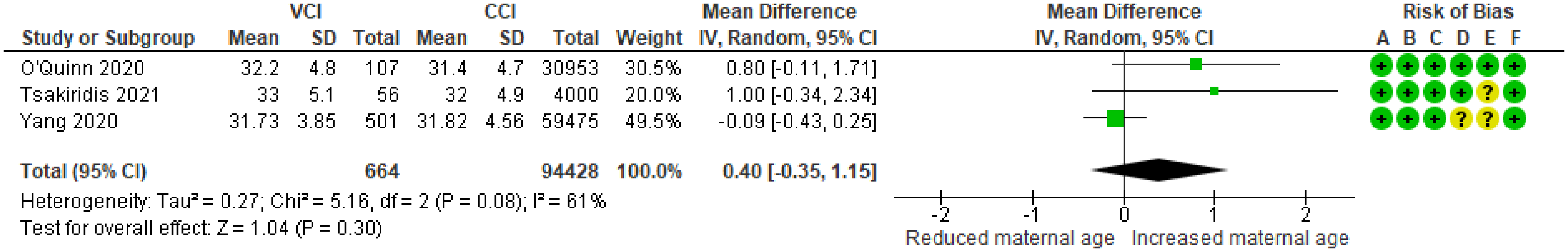


Abbreviations: CCI, central/eccentric cord insertion; CI, confidence interval; IV, weighted mean difference; SD, standard deviation; VCI, velamentous cord insertion

**Figure S3.** Forest plot demonstrating the risk for prenatally diagnosed VCI in singleton pregnancies relative to parity.

| Study or Subgroup | Nulliparity Events | Nulliparity Total | Multiparity Events | Multiparity Total | Weight | Risk Ratio M-H, Random, 95% CI | A | B | C | D | E | F |
|---|---|---|---|---|---|---|---|---|---|---|---|---|
| O'Quinn 2020 | 54 | 13227 | 42 | 13289 | 14.6% | 1.29 [0.86, 1.93] | + | + | + | + | + | + |
| Tsakiridis 2021 | 38 | 2524 | 18 | 1532 | 7.6% | 1.28 [0.73, 2.24] | + | + | + | + | ? | + |
| Yang 2020 | 238 | 24206 | 263 | 35770 | 77.7% | 1.34 [1.12, 1.59] | + | + | + | ? | ? | + |
| **Total (95% CI)** | | **39957** | | **50591** | **100.0%** | **1.33 [1.14, 1.55]** | | | | | | |
| Total events | 330 | | 323 | | | | | | | | | |

Heterogeneity: $Tau^2 = 0.00$; $Chi^2 = 0.04$, df = 2 (P = 0.98); $I^2 = 0\%$
Test for overall effect: Z = 3.59 (P = 0.0003)

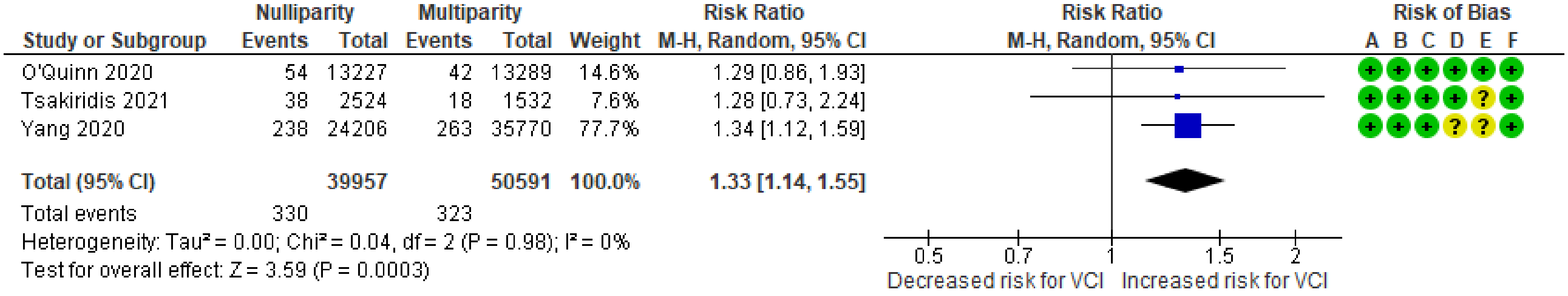


Abbreviations: CI, confidence interval; M-H, Mantel−Haenszel method; VCI, velamentous cord insertion

**Figure S4.** Risk of bias sensitivity analysis regarding ART - VCI association in singleton pregnancies.

| Study or Subgroup | ART Events | ART Total | Spontaneous conception Events | Spontaneous conception Total | Weight | Risk Ratio M-H, Random, 95% CI | A | B | C | D | E | F |
|---|---|---|---|---|---|---|---|---|---|---|---|---|
| Curtin 2019 | 11 | 16 | 111 | 453 | | Not estimable | ? | + | + | + | − | + |
| Ebbing 2016 | 589 | 15459 | 12422 | 795844 | 25.1% | 2.44 [2.25, 2.65] | + | + | + | + | + | + |
| Fukuda 2023 | 11 | 156 | 15 | 738 | 12.0% | 3.47 [1.62, 7.41] | + | + | + | + | ? | + |
| Gavriil 1993 | 6 | 51 | 1 | 62 | | Not estimable | − | + | + | + | ? | + |
| Jauniaux 1990 | 6 | 37 | 1 | 45 | | Not estimable | + | + | + | + | − | + |
| Larcher 2023 | 12 | 250 | 1 | 262 | 2.8% | 12.58 [1.65, 96.01] | + | + | + | + | + | + |
| O'Quinn 2020 | 5 | 1408 | 83 | 23926 | 9.9% | 1.02 [0.42, 2.52] | + | + | + | + | + | + |
| Raisanen 2012 | 22 | 677 | 611 | 26172 | 19.0% | 1.39 [0.92, 2.12] | + | + | + | + | ? | + |
| Tsakiridis 2021 | 9 | 175 | 47 | 3881 | 13.1% | 4.25 [2.12, 8.53] | + | + | + | + | ? | + |
| Visentin 2021 | 2 | 22 | 26 | 756 | | Not estimable | − | + | + | + | − | + |
| Yang 2020 | 19 | 1625 | 482 | 58351 | 18.1% | 1.42 [0.90, 2.23] | + | + | + | ? | ? | + |
| **Total (95% CI)** | | **19750** | | **909174** | **100.0%** | **2.14 [1.49, 3.08]** | | | | | | |
| Total events | 667 | | 13661 | | | | | | | | | |

Heterogeneity: $Tau^2 = 0.13$; $Chi^2 = 21.28$, df = 6 (P = 0.002); $I^2 = 72\%$
Test for overall effect: Z = 4.13 (P < 0.0001)

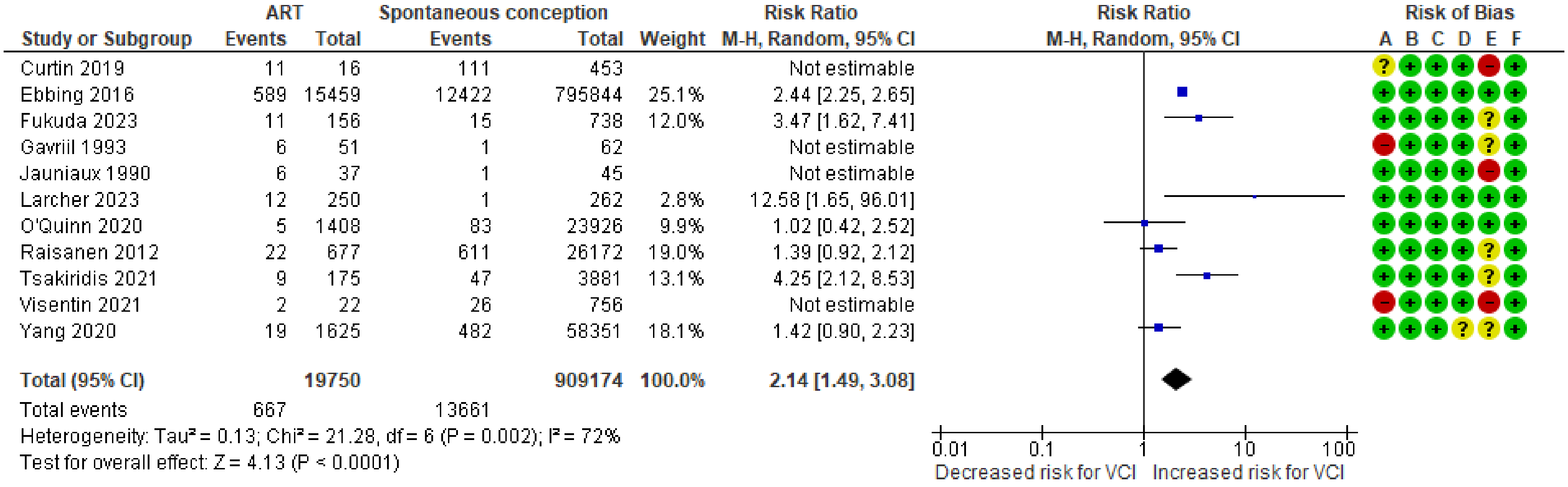


Abbreviations: ART, assisted reproductive technology; CI, confidence interval; M-H, Mantel−Haenszel method; VCI, velamentous cord insertion

**Figure S5.** Risk of bias sensitivity analysis regarding mean maternal age - VCI association in singleton pregnancies.

| Study or Subgroup | VCI Mean | VCI SD | VCI Total | CCI Mean | CCI SD | CCI Total | Weight | Mean Difference IV, Random, 95% CI | Risk of Bias A | B | C | D | E | F |
|---|---|---|---|---|---|---|---|---|---|---|---|---|---|---|
| Curtin 2019 | 29.05 | 6.23 | 122 | 28.55 | 6.29 | 347 | | Not estimable | ? | + | + | + | − | + |
| Hasegawa 2006 | 31 | 5.4 | 40 | 31.5 | 4.8 | 3367 | | Not estimable | + | + | + | + | − | + |
| Hasegawa 2009 | 33.1 | 3.4 | 13 | 32.8 | 5.2 | 487 | | Not estimable | + | + | + | + | − | + |
| O'Quinn 2020 | 32.2 | 4.8 | 107 | 31.4 | 4.7 | 30953 | 30.5% | 0.80 [-0.11, 1.71] | + | + | + | + | + | + |
| Sinkin 2017 | 30.5 | 6 | 53 | 29.4 | 5.7 | 103 | | Not estimable | − | + | + | + | ? | + |
| Tsakiridis 2021 | 33 | 5.1 | 56 | 32 | 4.9 | 4000 | 20.0% | 1.00 [-0.34, 2.34] | + | + | + | + | ? | + |
| Visentin 2021 | 35.57 | 6.24 | 28 | 33.39 | 5.5 | 750 | | Not estimable | − | + | + | + | − | + |
| Yang 2020 | 31.73 | 3.85 | 501 | 31.82 | 4.56 | 59475 | 49.5% | -0.09 [-0.43, 0.25] | + | + | + | ? | ? | + |
| **Total (95% CI)** | | | **664** | | | **94428** | **100.0%** | **0.40 [-0.35, 1.15]** | | | | | | |

Heterogeneity: Tau² = 0.27; Chi² = 5.16, df = 2 (P = 0.08); I² = 61%

Test for overall effect: Z = 1.04 (P = 0.30)

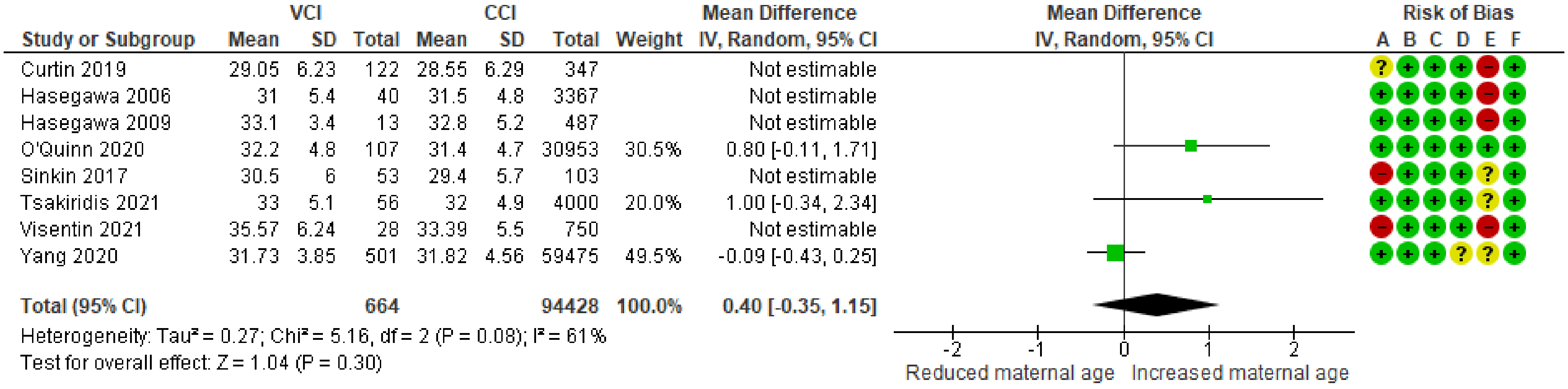


Abbreviations: CCI, central/eccentric cord insertion; CI, confidence interval; IV, weighted mean difference; SD, standard deviation; VCI, velamentous cord insertion

**Figure S6.** Risk of bias sensitivity analysis regarding nulliparity - VCI association in singleton pregnancies.

| Study or Subgroup | Nulliparity Events | Nulliparity Total | Multiparity Events | Multiparity Total | Weight | Risk Ratio M-H, Random, 95% CI | Risk of Bias A | B | C | D | E | F |
|---|---|---|---|---|---|---|---|---|---|---|---|---|
| Curtin 2019 | 57 | 234 | 65 | 235 | | Not estimable | ? | + | + | + | − | + |
| Ebbing 2016 | 5950 | 334832 | 7061 | 476471 | 91.0% | 1.20 [1.16, 1.24] | + | + | + | + | + | + |
| O'Quinn 2020 | 54 | 13227 | 42 | 13289 | 0.7% | 1.29 [0.86, 1.93] | + | + | + | + | + | + |
| Raisanen 2012 | 299 | 10890 | 334 | 15959 | 4.5% | 1.31 [1.12, 1.53] | + | + | + | + | ? | + |
| Sinkin 2017 | 29 | 76 | 24 | 80 | | Not estimable | − | + | + | + | ? | + |
| Tsakiridis 2021 | 38 | 2524 | 18 | 1532 | 0.3% | 1.28 [0.73, 2.24] | + | + | + | + | ? | + |
| Visentin 2021 | 17 | 381 | 11 | 395 | | Not estimable | − | + | + | + | − | + |
| Yang 2020 | 238 | 24206 | 263 | 35770 | 3.5% | 1.34 [1.12, 1.59] | + | + | + | ? | ? | + |
| **Total (95% CI)** | | **385679** | | **543021** | **100.0%** | **1.21 [1.17, 1.25]** | | | | | | |
| Total events | 6579 | | 7718 | | | | | | | | | |

Heterogeneity: Tau² = 0.00; Chi² = 2.73, df = 4 (P = 0.60); I² = 0%

Test for overall effect: Z = 11.42 (P < 0.00001)

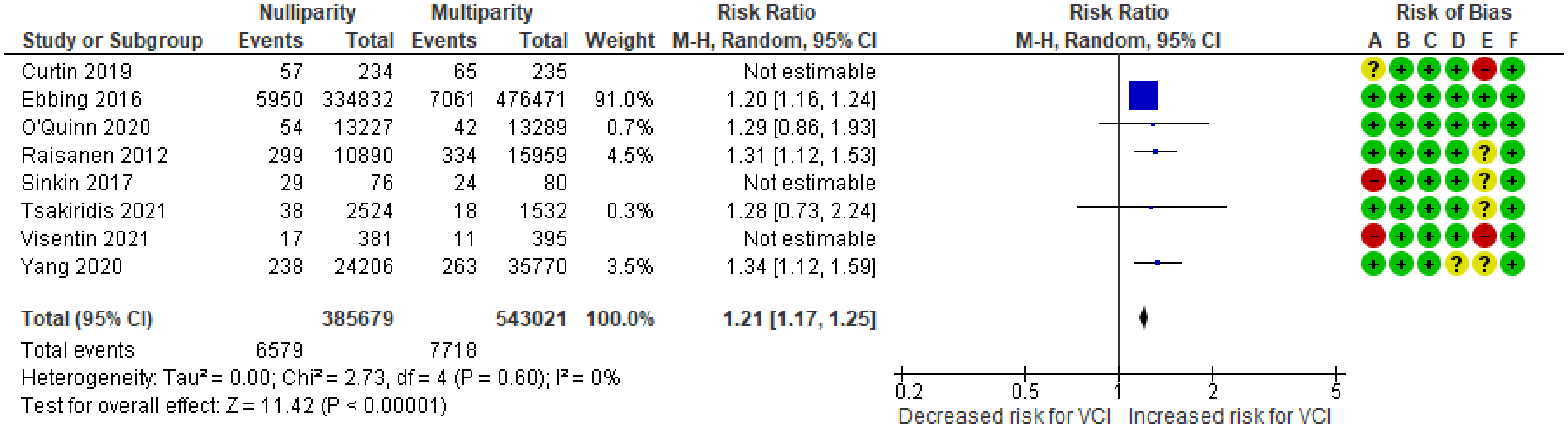


Abbreviations: CI, confidence interval; M-H, Mantel–Haenszel method; VCI, velamentous cord insertion

**Figure S7.** Risk of bias sensitivity analysis regarding smoking - VCI association in singleton pregnancies.

| Study or Subgroup | Smoking Events | Smoking Total | Not smoking Events | Not smoking Total | Weight | Risk Ratio M-H, Random, 95% CI | A | B | C | D | E | F |
|---|---|---|---|---|---|---|---|---|---|---|---|---|
| Curtin 2019 | 21 | 73 | 101 | 396 | 0.0% | 1.13 [0.76, 1.68] | ? | + | + | + | − | + |
| Ebbing 2013 | 1827 | 96151 | 6367 | 378869 | 80.5% | 1.13 [1.07, 1.19] | + | + | + | + | + | + |
| Heinonen 1996 | 15 | 762 | 201 | 11988 | 3.2% | 1.17 [0.70, 1.97] | + | + | + | + | ? | ? |
| O'Quinn 2020 | 6 | 2370 | 91 | 24487 | 1.3% | 0.68 [0.30, 1.55] | + | + | + | + | + | + |
| Raisanen 2012 | 37 | 1243 | 596 | 25606 | 7.7% | 1.28 [0.92, 1.77] | + | + | + | + | ? | + |
| Tsakiridis 2021 | 4 | 422 | 52 | 3634 | 0.9% | 0.66 [0.24, 1.82] | + | + | + | + | ? | + |
| Yang 2020 | 31 | 2529 | 470 | 57447 | 6.4% | 1.50 [1.04, 2.15] | + | + | + | ? | ? | + |
| **Total (95% CI)** | | **103477** | | **502031** | **100.0%** | **1.15 [1.05, 1.26]** | | | | | | |
| Total events | 1920 | | 7777 | | | | | | | | | |

Heterogeneity: Tau² = 0.00; Chi² = 5.38, df = 5 (P = 0.37); I² = 7%

Test for overall effect: Z = 2.91 (P = 0.004)

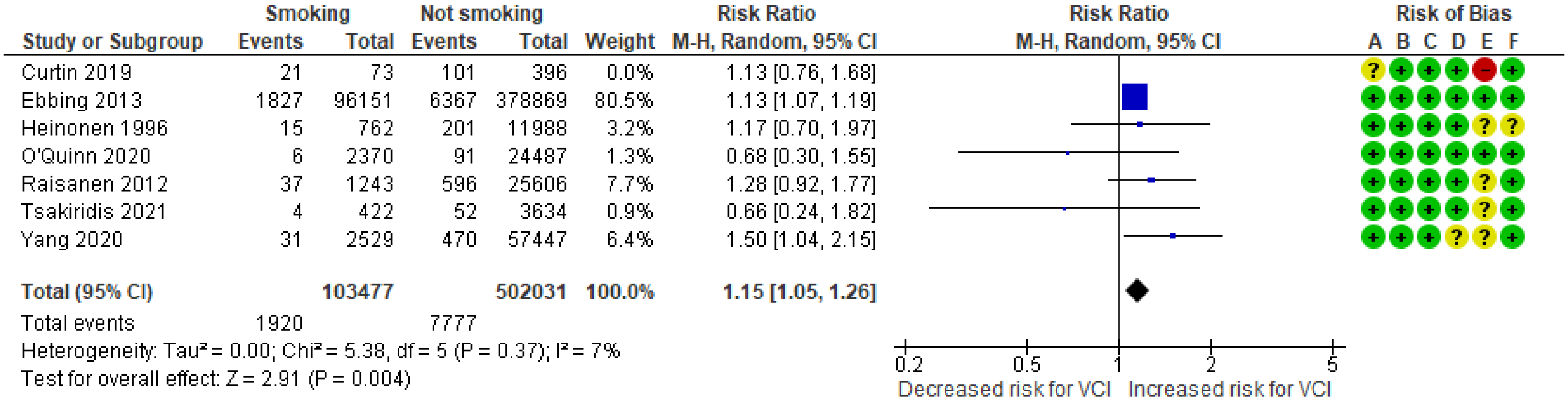


Abbreviations: CI, confidence interval; M-H, Mantel−Haenszel method; VCI, velamentous cord insertion

**Figure S8.** Risk of bias sensitivity analysis regarding prior cesarean section - VCI association in singleton pregnancies.

| Study or Subgroup | Prior cesarean section Events | Prior cesarean section Total | No prior cesarean section Events | No prior cesarean section Total | Weight | Risk Ratio M-H, Random, 95% CI | A | B | C | D | E | F |
|---|---|---|---|---|---|---|---|---|---|---|---|---|
| Ebbing 2015 | 2537 | 11407 | 99442 | 682995 | 28.6% | 1.53 [1.48, 1.58] | + | + | + | + | ? | + |
| Heinonen 1996 | 11 | 216 | 1336 | 12534 | 20.1% | 0.48 [0.27, 0.85] | + | + | + | + | ? | ? |
| Raisanen 2012 | 43 | 633 | 2055 | 26216 | 25.9% | 0.87 [0.65, 1.16] | + | + | + | + | ? | + |
| Sinkin 2017 | 8 | 53 | 16 | 103 | | Not estimable | − | + | + | + | ? | + |
| Yang 2020 | 36 | 501 | 4817 | 59475 | 25.4% | 0.89 [0.65, 1.22] | + | + | + | ? | ? | + |
| **Total (95% CI)** | | **12757** | | **781220** | **100.0%** | **0.91 [0.57, 1.46]** | | | | | | |
| Total events | 2627 | | 107650 | | | | | | | | | |

Heterogeneity: Tau² = 0.20; Chi² = 40.86, df = 3 (P < 0.00001); I² = 93%

Test for overall effect: Z = 0.39 (P = 0.70)

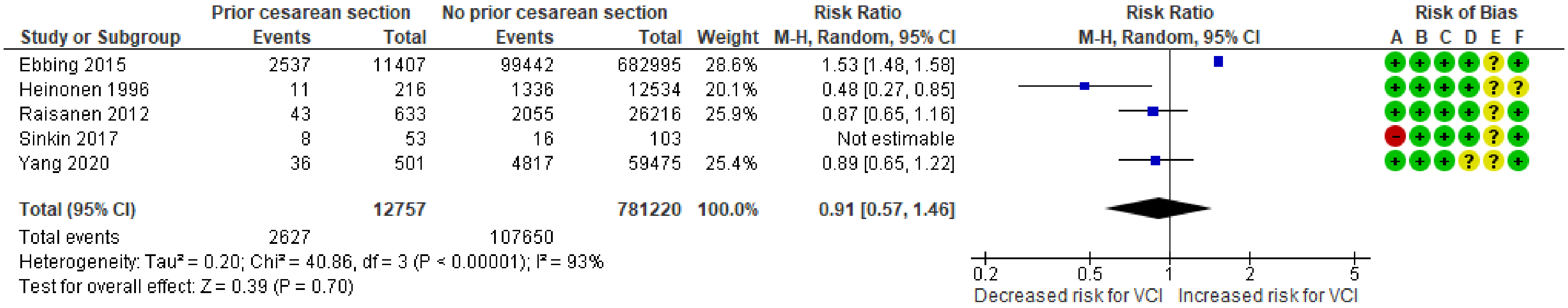


Abbreviations: CI, confidence interval; M-H, Mantel−Haenszel method; VCI, velamentous cord insertion

**Figure S9.** Risk of bias sensitivity analysis regarding placenta previa - VCI association in singleton pregnancies.

| | Placenta previa | | Control group | | | Risk Ratio | Risk of Bias | | | | | |
|---|---|---|---|---|---|---|---|---|---|---|---|---|
| Study or Subgroup | Events | Total | Events | Total | Weight | M-H, Random, 95% CI | A | B | C | D | E | F |
| Ebbing 2013 | 102 | 1614 | 9398 | 572461 | 79.5% | 3.85 [3.19, 4.65] | + | + | + | + | + | + |
| Heinonen 1996 | 3 | 53 | 213 | 12697 | 2.9% | 3.37 [1.12, 10.21] | + | + | + | + | ? | ? |
| Raisanen 2012 | 11 | 195 | 622 | 26654 | 10.3% | 2.42 [1.35, 4.31] | + | + | + | + | ? | + |
| Visentin 2021 | 1 | 7 | 27 | 771 | 0.0% | 4.08 [0.64, 26.00] | − | + | + | + | − | + |
| Yang 2020 | 8 | 365 | 493 | 59611 | 7.3% | 2.65 [1.33, 5.29] | + | + | + | ? | ? | + |
| **Total (95% CI)** | | **2227** | | **671423** | **100.0%** | **3.56 [2.94, 4.30]** | | | | | | |
| Total events | 124 | | 10726 | | | | | | | | | |

Heterogeneity: Tau² = 0.00; Chi² = 3.09, df = 3 (P = 0.38); I² = 3%
Test for overall effect: Z = 13.18 (P < 0.00001)

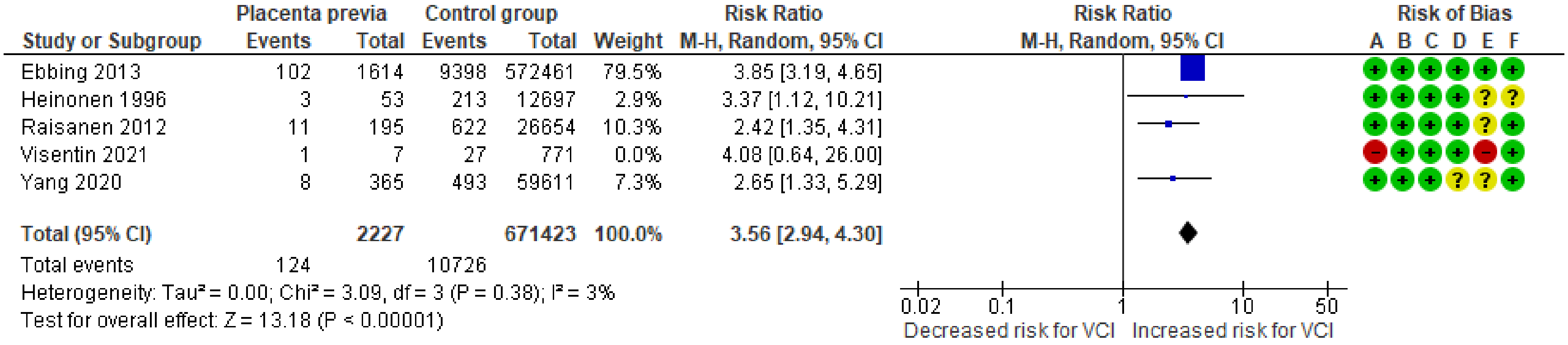


Abbreviations: CI, confidence interval; M-H, Mantel−Haenszel method; VCI, velamentous cord insertion

**Figure S10.** Risk of bias sensitivity analysis regarding chronic hypertension - VCI association in singleton pregnancies.

| | Chronic hypertension | | Control group | | | Risk Ratio | Risk of Bias | | | | | |
|---|---|---|---|---|---|---|---|---|---|---|---|---|
| Study or Subgroup | Events | Total | Events | Total | Weight | M-H, Random, 95% CI | A | B | C | D | E | F |
| Aragie 2022 | 4 | 44 | 8 | 368 | 26.1% | 4.18 [1.31, 13.32] | + | + | + | + | ? | + |
| Ebbing 2013 | 70 | 3121 | 9430 | 571954 | 61.7% | 1.36 [1.08, 1.72] | + | + | + | + | + | + |
| O'Quinn 2020 | 1 | 330 | 106 | 30614 | 12.2% | 0.88 [0.12, 6.25] | + | + | + | + | + | + |
| Sinkin 2017 | 3 | 7 | 50 | 149 | 0.0% | 1.28 [0.53, 3.09] | − | + | + | + | ? | + |
| **Total (95% CI)** | | **3495** | | **602936** | **100.0%** | **1.73 [0.81, 3.70]** | | | | | | |
| Total events | 75 | | 9544 | | | | | | | | | |

Heterogeneity: Tau² = 0.23; Chi² = 3.71, df = 2 (P = 0.16); I² = 46%
Test for overall effect: Z = 1.40 (P = 0.16)

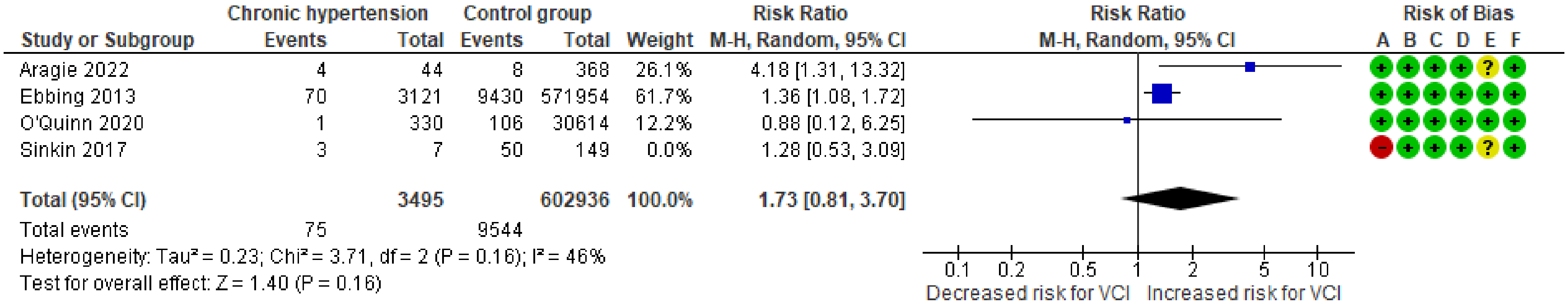


Abbreviations: CI, confidence interval; M-H, Mantel−Haenszel method; VCI, velamentous cord insertion
